# Sex determination systems as the interface between male-killing bacteria and their hosts

**DOI:** 10.1098/rspb.2021.2781

**Published:** 2022-04-13

**Authors:** Emily A. Hornett, Daisuke Kageyama, Gregory D. D. Hurst

**Affiliations:** ^1^ Institute of Infection, Veterinary, and Ecological Sciences, University of Liverpool, Biosciences Building, Liverpool L69 7ZB, UK; ^2^ Vector Biology, LSTM, Liverpool L3 5QA, UK; ^3^ Institute of Agrobiological Sciences, NARO, Tsukuba, Japan

**Keywords:** male killing, resistance, sex ratio, endosymbionts

## Abstract

Arthropods host a range of sex-ratio-distorting selfish elements, including diverse maternally inherited endosymbionts that solely kill infected males. Male-killing heritable microbes are common, reach high frequency, but until recently have been poorly understood in terms of the host–microbe interaction. Additionally, while male killing should generate strong selection for host resistance, evidence of this has been scant. The interface of the microbe with host sex determination is integral to the understanding of how death is sex limited and how hosts can evolve evasion of male killing. We first review current knowledge of the mechanisms diverse endosymbionts use to induce male-specific death. We then examine recent evidence that these agents do produce intense selection for host nuclear suppressor elements. We argue, from our understanding of male-killing mechanisms, that suppression will commonly involve evolution of the host sex determination pathways and that the host's response to male-killing microbes thus represents an unrecognized driver of the diversity of arthropod sex determination. Further work is required to identify the genes and mechanisms responsible for male-killing suppression, which will both determine the components of sex determination (or other) systems associated with suppressor evolution, and allow insight into the mechanism of male killing itself.

## 1. Background

Maternally inherited endosymbionts are remarkably common in arthropods and have evolved an array of manipulations of host reproduction to favour their transmission through the female line. These include microbes that distort the host sex ratio by selectively killing male offspring (male killing, MK). As vertically transmitted microbes are generally exclusively maternally inherited, the male is an evolutionary dead end and thus male death will not impede the symbiont's spread. Indeed, MK may indirectly enhance spread where it benefits the dead males' infected female siblings relative to uninfected females, for example through sibling egg consumption, a reduction in sibling competition or a reduction in inbreeding [1].

Despite MK being discovered in the early twentieth century, it was not until 2006 that host suppression of MK was observed, in the butterfly *Hypolimnas bolina* [2]. This is perhaps surprising as the strength of selection for suppression arising from MK is among the strongest possible. First, there is the impact of their action on the individual that carries them: killing males results in a ca. 50% fitness loss to the female bearer. Second, when male-killers become common, the selection for suppression increases in an accelerating fashion. This is because a common male-killer produces a skewed population sex ratio, and when males are rare, Fisherian selection [3] for their production is intense, and this is then reflected in intense selection to restore male production.

MK and suppression of MK are very likely to be mechanistically linked through the process of host sex determination. MK is a form of sex-specific lethality that will commonly involve either direct interference with sex determination processes or a cue from sex determination as to host gender. Suppression of MK, conversely, will commonly involve change(s) in those systems to avoid MK. In this review, we first outline mechanistic studies that have begun to elucidate the means by which MK functions. We then examine our current knowledge of cases of suppression. We argue this mechanistic understanding is key in allowing us to now consider not just why suppression evolves, but also the genetic systems that may be involved, and how changes of these impact our understanding of arthropod evolution. We conclude by discussing whether sex-ratio-distorting microbes may be important drivers of arthropod sex determination diversity.

## 2. The mechanisms of MK: case studies

Male-killers are diverse (electronic supplementary material, table S1) and include bacterial strains or species from genera such as *Wolbachia* and *Rickettsia* (Alphaproteobacteria), *Spiroplasma* (Mollicutes), Flavobacteria (Flavobacteria) and *Arsenophonus* (Gammaproteobacteria), but also certain RNA viruses. Within a particular bacterial genus, MK may have evolved independently several times; for instance, *Spiroplasma* strains from distantly related clades (e.g. *S. ixodetis* and *S. poulsonii*) can cause MK. Additionally, MK strains can be closely related to non-MK strains. Male-killers are in turn hosted by a wide range of arthropods with divergent sex determination systems: MK has been observed in Lepidoptera (ZW female heterogamety), flies, lacewings and beetles (generally XY male heterogamety), a pseudoscorpion (male XO heterogamety), and hymenopteran wasps (haplodiploidy). The complex diversity in hosts and symbionts leads us to question whether phylogenetically divergent male-killers adopt similar methods of targeting and killing males, and how one male-killer induces MK in hosts with divergent sex determination systems.

### (a) MK *Spiroplasma*

The pathology of MK caused by *Spiroplasma poulsonii* in *Drosophila* flies is fairly well known: mortality occurs during embryogenesis, through delayed and abnormal male embryo development [4], extensive cellular apoptosis in male embryos [5,6] and severe disruption of male nervous tissue [7]. However, until recently little was understood of how *Spiroplasma* specifically targets males. In *Drosophila*, expression of the female-specific developmental switch gene, *Sex-lethal* (*Sxl*), establishes whether the zygote develops as a female or male. While MK *Spiroplasma* does not alter *Sxl* expression itself (in *D. nebulosa* [6]) or influence somatic sexual identity (in *D. melanogaster* [8]), it has become evident that MK *Spiroplasma* does interact with the host's dosage compensation system. Dosage compensation acts to equalize transcript levels of the X chromosome between males (XY) and females (XX) by upregulating gene expression on the single male X chromosome, and is mediated by a ribonucleoprotein complex: the dosage compensation complex (DCC), aka the male-specific lethal (MSL) complex. In *D. melanogaster*, *Spiroplasma* is only able to induce MK when all five proteins in the complex are present and fully functional [9].

Further work demonstrated that *Spiroplasma* can target and damage DNA on the dosage-compensated male X chromosome that interacts with the functional DCC. The damaged male X chromosome exhibits chromosomal bridge formation and breakage, which in turn triggers massive abnormal apoptosis via the host's p53-dependent pathways [10]. The DCC also becomes mis-localized prior to male death. Tellingly, when the DCC was artificially formed in infected females through transgenic expression of MSL2 (normally only expressed in males), mis-localization of the DCC and female death occurred [11].

Genomic comparison of MK *Spiroplasma* variants naturally carried by *D. melanogaster* revealed that expression of one gene is sufficient for MK activity. In a fully penetrant (all males die) MK strain, the plasmid gene *spaid* (*S. poulsonii* androcidin) encodes a 1065aa ankyrin-repeat protein, while in a less penetrant MK strain the *spaid* locus contains a single amino acid substitution as well as an 828 bp deletion. Ectopic expression of *spaid* as a C-terminal GFP fusion protein in *D. melanogaster* recapitulated the natural MK pathologies. In addition, *spaid* expression in transgenic females engineered to express the DCC triggered massive apoptosis, confirming that, in transgenic systems at least, *spaid* mediates its effects through the dosage compensation machinery [12].

### (b) MK *Wolbachia*

In *Drosophila bifasciata,* MK *Wolbachia* produce similar pathological responses in the host to that induced by MK *Spiroplasma* in *D. melanogaster*, including triggering male-specific abnormal apoptosis, and inducing DNA damage and chromatin bridges on the dosage compensated male X chromosome. MK *Wolbachia*-infected male *D. bifasciata* embryos also exhibit defective chromatin remodelling and chromosome segregation specifically on the dosage-compensated male chromosome [13]. However, in contrast to MK *Spiroplasma* infected flies, the neural development of male flies infected with MK *Wolbachia* proceeds normally, and the dosage compensation complex is not mis-localized [14].

The candidate gene for MK in *Wolbachia* is *wmk* (*WO-mediated killing*), a putative transcriptional regulator encoded on prophage WO [15]. In contrast to *spaid*, evidence for *wmk*'s involvement in MK relies predominantly on transgenic expression. However, expression of *wmk* in transgenic *D. melanogaster* consistently recapitulated the cytological defects and DNA damage seen in naturally MK-*Wolbachia*-infected male flies. In an investigation of the impact of *wmk* genetic variation upon MK, one codon was found to segregate among homologs from divergent hosts by the phenotype expressed. Functional testing of synonymous variation in this Serine codon revealed that a single silent nucleotide change in *wmk* impacts presence/absence of MK in transgenic lines. Changes in the predicted mRNA secondary structure or other post-transcriptional modifications of *wmk* are thought to underpin the phenotypic variation observed [16].

In contrast to *Drosophila* (male heterogamety), most butterflies and moths (Lepidoptera) exhibit female heterogamety (females ZW and males ZZ). In the moth *Bombyx mori*, femaleness is determined by the W chromosome, which encodes a dominant feminizing gene (*Fem*) [17,18]. *Fem* is a precursor of a single W-derived PIWI-interacting RNA (piRNA) that targets *Masculinizer* (*Masc*), a gene on the Z chromosome. *Masc* is a lepidopteran-specific zinc finger protein gene required for both masculinization and dosage compensation. Towards the end of the sex determination cascade the transcript of the core sex-determining gene, *doublesex* (*dsx*), is sex-specifically spliced into female and male forms of mRNA. Depletion of *Masc* in male embryos produces the default female-type splicing of *dsx*. Furthermore, silencing of *Masc* causes male-specific lethality as the *Masc* protein is required for the repression of global transcription (dosage compensation) from the Z chromosome in male embryos [18].

In *Ostrinia* moths, *Wolbachia*-induced MK occurs during late embryogenesis or at the first-instar larval stage, where growth is retarded but there are no discernible morphological abnormalities [19]. Here, *Wolbachia* specifically kills males by downregulating *Masc* expression thereby preventing proper dosage compensation of Z-linked genes in male somatic tissue. The role of *Masc* in MK was confirmed in *O. furnacalis*, in which injection of *in vitro* transcribed *Masc* to *Wolbachia*-infected embryos rescued males [20]. Unusually, in both *O. furnacalis* and *O. scapulalis*, while presence of *Wolbachia* results in MK, elimination of *Wolbachia* leads to the death of females while rescuing males. In naturally uninfected males and females, ZZ males and ZW females carry the male- or female-specific isoforms of *dsx*, respectively. However, in lines that have co-evolved with *Wolbachia*, all *Wolbachia*-infected individuals, regardless of genetic sex, express the female *dsx* (and males die). Conversely, both male and female offspring of females cured of *Wolbachia* (usually through antibiotics), express the male *dsx* (and females die) [19]. It appears that *Wolbachia* is intrinsically associated with the moth's sex determination system, and infection, or the removal of infection, results in discordance between the genetic and phenotypic sex, and ultimately death.

One important characteristic of ZW female-heterogametic animals is that they have a female-linked W chromosome. Being maternally inherited, cytoplasmic symbionts are co-inherited with the W chromosome and so share evolutionary dynamics. It has been speculated that the putative female-determining function encoded on the W chromosome has been lost in *Wolbachia*-infected *Ostrinia* females, driven by the presence of a *Wolbachia* strain that also had a female-determining factor. ZW embryos produced by *Wolbachia*-eliminated females are thus masculinized and die. The dying ZW individuals exhibit improper dosage compensation of Z-linked genes, whose expression level is nearly half that of normal ZW and ZZ [19,21]. In *Wolbachia*-infected matrilines, therefore, females require the presence of *Wolbachia*. Although curing other lepidopteran species of MK infections does not have the same effect, degradation of W chromosome function, or even the loss of the entire W chromosome, might be an inevitable fate of the long-term evolutionary relationships between MK symbionts and lepidopteran hosts, as has been shown in the feminizing *Wolbachia*/*Eurema* butterfly interaction [22].

### (c) MK *Arsenophonus*

The gammaproteobacterium *Arsenophonus nasoniae* is currently only known to induce MK in the parasitic jewel wasp *Nasonia vitripennis* (‘son-killer’ [23–25]). *Nasonia vitripennis* has haplodiploid sex determination, where fertilized eggs develop as diploid females and unfertilized eggs develop parthenogenetically as haploid males. *Arsenophonus* also differs from *Wolbachia* and *Spiroplasma* in that it resides solely intercellularly within somatic tissue, is not present inside eggs, and instead has a pronounced tropism for the ovipositor enabling vertical transmission [26]. The MK mechanism employed by *Arsenophonus* takes advantage of the sex-specific inheritance pattern of the centrosome associated with haplodiploidy, and mortality occurs before the processes associated with sex determination are enabled. The centrosome is an organelle found near the nucleus in the cell cytoplasm, and is important in microtubular organization, and cell polarity and division. In most diploid organisms the centrosome is derived from the sperm, however in haplodiploid species development of haploid (unfertilized) male eggs has necessitated the formation of a maternal centrosome [27,28]. While maternally derived centrosomes also form in fertilized female embryos, they degenerate and only paternally derived centrosomes are retained [29]. Significantly, fertilized zygotes that become secondarily haploid (e.g. through the action of PSR supernumerary chromosomes [30]) are not killed, establishing the importance of fertilization protecting against death, rather than ploidy and sexual identity *per se*, in the MK process. While the method by which *Arsenophonus* prevents formation of the maternal centrosome is unknown, the extracellular lifestyle of *Arsenophonus* is consistent with a diffusible toxin that effects this inhibition [31]. Recent work has detected many toxin elements within the *Arsenophonus* genome, although no genes with similarity to either *spaid* or *wmk* [32].

While work on the mechanism of MK is limited to three microbes in five host genera, key conclusions can nevertheless be drawn. First, MK is one phenotype with multiple genetic causes in terms of ‘toxin’ genes. Second, different male-killers act to produce sex-specific mortality through interacting with different aspects of male/female biology. MLS is associated with the presence/absence of fertilization (*Arsenophonus* in *Nasonia*), activity of dosage compensation machinery (*Spiroplasma* in *Drosophila*) and interference with core sex determination genes (such as *Wolbachia* mediated changes in *doublesex* in *Ostrinia*). From this understanding of the mechanism of MK, one can then examine the evolutionary biology of suppression.

## 3. Host resistance of MK: case studies

While theory states that MK should impose strong selection upon the host to evolve a resistance mechanism, particularly when MK bacteria are at high prevalence, demonstrable evidence of resistance has until recently been elusive. There are three potential methods of resistance: the host could (1) directly kill the symbiont, (2) reduce the symbiont's transmission or (3) evolve suppression of the MK action of the symbiont, allowing infected males to survive. Evidence of the first is particularly hard to obtain as once the symbiont is killed, there may be no trace of previous infection. Similarly, transmission suppressors are inconspicuous as they remove or reduce the presence of the symbiont. There is also the complication with deciphering the difference between presence of a selected allele that prevents transmission of MK versus a host genetic background that isn't suitable for the infection to persist (i.e. is it an evolved response to infection or an incompatibility?). In all three cases, resistance against sex-ratio distorters is hard to identify; as soon as resistance spreads, the bias lessens and commonly disappears. That said, evidence of host suppression of MK has accrued over the last 15 years through observation of changes in population sex ratio, from breeding studies identifying segregation of genes associated with male survival (introgression) and following transfer of symbionts between hosts (trans-infection) (electronic supplementary material, table S2).

### (a) Observation of host suppressor evolution in natural populations

Rapid evolution of host suppression of MK activity has been observed in natural populations of two systems: *H. bolina* butterflies/*Wolbachia* and *Mallada desjardinsi* lacewings/*Spiroplasma*. In both cases, a host population that initially exhibited high frequency of a male-killer and thus was highly female-biased, was observed to transform over only a few years to one with a sex ratio at parity [2,33–35].

Female-biased sex ratios have been noted in *H. bolina* since the 1920s (e.g. [36]). Later work identified this to be due to the death of immature males [37], caused by *Wolbachia* (strain *w*Bol1) prevalent across much of the butterflies' range [38]. *Hypolimnas bolina* is remarkable for its propensity to carry MK *Wolbachia* at extreme frequencies. In 2001, a study of *H. bolina* in Samoa revealed that 99% of females were *w*Bol1-infected, resulting in a population sex ratio of 1 male per 100 females. Historical records indicate that this severely biased sex ratio had persisted for greater than 100 years, equating to approximately 400 butterfly generations [33]. Observation in 2005 of a rapid increase in the number of males led to the discovery of a host suppressor of MK action that by 2006 had restored the sex ratio to near parity [2]. This shift in population sex ratio in fewer than 10 generations supports the prediction that populations at high MK frequency are under intense selection for suppression. Juxtaposed against this is the observed protracted period of extreme sex ratio bias prior to suppressor evolution. Together this may indicate that while a host suppressor can spread rapidly once it has entered the population, *in situ* mutations that stop MK action experience some constraint.

Genetic analysis of museum samples of this butterfly allowed the phenotype and prevalence of *w*Bol1 in historical populations of *H. bolina* to be inferred. During the late 1800s to 1910s, all sampled males and females from the Philippines were infected with *w*Bol1, suggesting that a MK suppressor existed over 100 years ago in this area, as it does today. During the same period no infected males were found in neighbouring Malaysian Borneo, despite *w*Bol1 being at high frequency in females, a condition that persisted until the late 1960s [37]. Recent sampling shows that the suppressor is now present in Borneo. At the eastern margin of the butterfly's range (Fiji and French Polynesia), there is no evidence, past or present, of host suppression of MK [39].

This inter-population polymorphism of the phenotype of *w*Bol1 allowed the role of host nuclear suppressor genes to be tested by placing MK and non-MK *w*Bol1 isolates against the alternate host genetic background. Female butterflies infected with MK *w*Bol1 (suppressor genotype ss) were crossed to *w*Bol1-infected males carrying a suppressor (SS). After only one generation (offspring with Ss genotype) the MK *w*Bol1 failed to kill all males. It took two generations of the reciprocal cross of *w*Bol1-infected females (SS) with uninfected males (ss) to ‘remove’ sufficient genetic material (i.e. two copies of the suppressor locus) before MK occurred. In both cases, one generation of backcross reverted the phenotype. This led to the conclusion that the MK suppressor in *H. bolina* is zygotically acting, dominant and autosomal [40]. This work also shows that where *w*Bol1 does not cause MK due to the presence of the suppressor, it retains MK ability. Interestingly, the suppressed MK *w*Bol1 induces a second reproductive manipulation common to *Wolbachia*, Cytoplasmic Incompatbility (CI) through the surviving infected males: *w*Bol1-infected males do not produce viable offspring when mated with uninfected females [41].

Data from the introgression experiments described above are compatible with the suppressor being controlled at a single locus. Further investigation revealed that only one genomic region is necessary for males to survive *w*Bol1-induced MK [42]. Uniquely in this system, data of the suppressor locus has been combined with the real-time observation of natural selection in Samoa, to allow study of the genomic impact of the rapid spread of the suppressor. The pattern of genetic variants across the chromosomal region containing the suppressor was examined in butterflies collected pre- and post-suppressor spread. The genomic imprint was remarkably large, with allele frequencies having changed across a 25cM region surrounding the suppressor locus during the selective sweep [43].

The presence of novel associated alleles in post-spread samples, and concordance in the position of the suppressor in butterflies from south east Asia with the genomic region under selection in Samoa, support the premise that the suppressor arrived in an immigrant butterfly rather than through *de novo* mutation. Future research in this system should aim to identify the nature of the suppressor mutation itself and to that end a strong candidate gene has been found within the region of interest: *doublesex* (*dsx*), the core sex-determining gene in Lepidoptera [43], which as we have seen is also involved in the *Ostrinia* moth/MK *Wolbachia* interaction [19].

A similar case has since been observed in the green lacewing *M. desjardinsi*. Here, a female-biased population sex ratio (57 females: 7 males) was first observed in 2011 in Matsudo, Chiba Prefecture, in central mainland Japan [34]. At this time, 71% of females tested were *Spiroplasma*-infected and the majority of infected females gave rise to all-female progeny, with nearly half of each brood having died during the embryonic or first-instar larval stage. *Spiroplasma* was confirmed as the MK agent, and the sex ratio could be restored to 1 : 1 by curing infected females of *Spiroplasma* with antibiotics. Interestingly, four *Spiroplasma*-infected females produced male offspring, suggesting that despite being low in frequency, suppressors against MK were already present in 2011. Although the sex ratio bias produced by individual females was less severe than in *H. bolina* (10% versus 1% male), continued selection for MK suppression was anticipated.

In 2016, 5 years after the first observation, the sex ratio of the same population was less skewed (80 females and 49 males). In this sample, 52% of females tested were infected with *Spiroplasma* but all produced both male and female offspring. Crossing these normal sex ratio females that were assumed to carry a MK suppressor, with males derived from laboratory stock that was established from insects collected in 2011, resulted in the re-appearance of the MK phenotype [35]. The detailed mode of inheritance remains unclear in the *M. desjardinsi* system.

### (b) Host suppressors revealed during nuclear introgression experiments

Polymorphism in MK has been observed in several insects including *Drosophila*, ladybirds and planthoppers. This variation may be due to (a) different symbiont isolates expressing MK to different degrees, (b) the symbiont and/or MK phenotype being sensitive to variation in host physiology or environment, or (c) variation in host genetic factors that suppress or reduce MK. Perhaps the very earliest indication of host resistance of cytoplasmic MK occurred in a study of *Drosophila prosaltans* in the 1950s where crosses between MK and non-MK lines suggested the presence of a recessive suppressor [44].

In two more recent case studies, nuclear introgression crosses were used to test whether host genotype affected variation in MK expression. MK bacteria are particularly common in ladybird beetles [45] where they are occasionally found at high frequency [46,47]. Despite several systems being rigorously studied, host resistance has only been characterized in *Cheilomenes sexmaculata*, which harbours a MK gammaproteobacterium. Here, MK-infected females from two lines originating in Tokyo were crossed to males from non-MK (normal sex ratio) lines. Unexpectedly, the nature of the MK trait depended on which male the female had mated with. The crossing data indicated the presence of a polymorphic autosomal dominant Mendelian inherited gene that suppresses MK activity rather than transmission [48].

The second study concerns the recent discovery of MK suppression in the planthopper *Laodelphax striatellus*. MK in this system is caused by *Spiroplasma* *ixodetis* and is unusual in that infected male offspring die in later nymphal stages rather than during embryogenesis or as early instar larvae. During continuous rearing of *L. striatellus*, one line produced males despite being infected. The presence of a suppressor was tested by serially introgressing genetic material from uninfected males (lacking the suppressor) into the ‘suppressed’ female line. Two generations of crosses were required to restore MK to this line, and only one backcross was necessary to rescue males, revealing the presence of a zygotic dominant suppressor [49].

### (c) Host suppression indicated following interspecies trans-infection

Commonly, trans-infection experiments are deployed to ascertain the influence of host genetic background on symbiont phenotype. Here, bacteria are transferred to a novel host species (or background) through microinjection, or if the species are closely related, hybridization (reviewed in [50]). MK can notably appear or disappear in the recipient host following trans-infection, suggesting the presence of host resistance genes in the donor or recipient species, respectively.

In the first study, CI-inducing *Wolbachia* were transferred from their native *Drosophila recens* into two strains of a naturally uninfected sister species *D. subquinaria* via hybridization and serial backcrossing. While there was no evidence of MK in the Alberta strain of *D. subquinaria*, essentially complete MK occurred in *D. subquinaria* from Vancouver. Additionally, the offspring sex ratio of Vancouver females carrying the novel MK infection depended on male partner identity: when mated to Vancouver males, near complete MK was observed. However, backcrosses to Alberta *D. subquinaria* or *D. recens* males reduced the penetrance of MK and males survived. The data indicated that the MK suppressor apparently present in *D. recens* is dominant, zygotic, autosomal, and is most probably governed by multiple loci rather than being a single-locus effect. Susceptibility to MK was tested in further strains of *D. subquinaria*, and MK was observed several times. Resistance (and hence susceptibility) to MK is, therefore, polymorphic in this species [51].

In a second study, MK again emerged following transfer of *Wolbachia*. The almond moth *Ephestia (Cadra*) *cautella* is doubly infected with two *Wolbachia* strains (*w*CauA and *w*CauB) and expresses CI, while the Mediterranean flour moth *Ephestia kuehniella* harbours one partially CI-inducing *Wolbachia* strain, *w*Kue. *Wolbachia* derived from *E. cautella* were transferred to cleared (uninfected) *E. kuehniella*. Lines infected with *w*CauA, either singly or when co-infected with *w*CauB, expressed complete MK in contrast to the strong CI observed in its native *E. cautella*. These data suggest *E. kuehniella* is susceptible to MK while *E. cautella* is not, and thus may carry host suppressors of MK. Interestingly, reports from the 1970s are consistent with the presence of active MK in *E. cautella*, which implies the spread of suppression may be recent [52–54].

In a third study, the presence of host suppressors was tested in *D. melanogaster* infected with *Spiroplasma* strain NSRO, originally derived from *D. nebulosa*. Among 10 lines studied, crosses to males of two lines (Sevelen and Hikone) attenuated the intensity of MK, suggesting the presence of host factors that suppress MK. Corroborating these data, trans-infection (by microinjection) of MK *Spiroplasma*-laden haemolymph into uninfected females of the Sevelen and Hikone lines similarly resulted in reduced expression of MK. The mating schemes employed revealed that the suppressors of MK activity found in the Sevelen and Hikone lines are maternal and dominant. In Sevelen they are mainly located on the X chromosome whereas in Hikone they are on autosomes, indicating independent origins [55].

It is clear that suppression of MK has evolved a number of times. In addition to direct observation of suppression evolution in two cases, there are now studies evidencing the presence of polymorphic suppression or the release of MK upon trans-infection that implies fixed suppression. Importantly, the evidence of variation in suppression in species that do not naturally carry a MK symbiont implies that novel MK symbionts (arriving through a host shift) may do so in the context of standing genetic variation for suppression, that has evolved for reasons distinct from suppression.

## 4. Should we always expect suppressors of MK to evolve?

The above studies demonstrate that suppression occurs widely, but the frequency with which suppression evolves is unclear. Host suppressors have been sought but not found in only a handful of cases including MK *Wolbachia* infecting *D. bifasciata* [56], *D. innubila* [57] and the butterfly *Acraea encedon* [58] and for MK *Spiroplasma* infecting *D. melanogaster* [59]. However, there are clearly biases against reporting negative results.

Modelling approaches allow us some insight into the factors that determine whether or not suppression can spread [60]. The conditions for suppressor invasion are determined by the balance of the benefits of suppression in terms of rescuing males, and the costs of carrying a suppressor mutation. There may exist a cost of carrying the suppressor for uninfected individuals (males or females), which manifests as a reduction in the performance of the carrier through either a metabolic cost associated with expression of the suppressor or through suppression altering key systems (such as male/female development) away from their optima. There may also be a cost associated with the relative performance of rescued males compared to uninfected males. The magnitude of these costs remain theoretical—measurement has not been attempted in any system.

The conditions for suppressor invasion are commonly broad because (a) MK itself produces a major fitness loss, with half of the progeny of an infected female dying. Even at 1% male-killer prevalence, the selective coefficient for a cost-free suppressor is 0.005, a value that would generally be regarded as ‘strong selection’. In addition, (b) as MK frequency increases, this generates further Fisherian selection for the suppressor [3]. This arises as increasing MK frequency makes the population sex ratio progressively more female biased, and the expected fertility of rescued males thus increases. Intense selection for suppression in high prevalence MK populations has been witnessed directly in *H. bolina* and *M. desjardinisi*, where the rapid spread of suppression has been recorded in real time [2,35,40].

One important caveat is that the situation alters where the symbiont can additionally induce CI, as is the case for the *Wolbachia* strain *w*Bol1 in *H. bolina* [41] and *w*CauA in *E. cautella* [54]. Where the MK strain can secondarily induce CI through rescued males, conditions for invasion of the suppressor become much more restrictive when the symbiont exists at low prevalence [61]. This is because the rescued male, carrying the suppressor, does not form viable zygotes when mated with the majority of females in the population (the uninfecteds), and thus spread of the suppressor is impeded.

Overall, the invasion conditions for a suppressor will depend on the frequency of MK before suppressor invasion, the cost of carrying the suppressor in infected and uninfected individuals, and the presence of CI through rescued males. Costly suppression can prevent invasion of suppression for MK at low initial prevalence, and the CI phenotype in rescued males can prevent invasion for MK up to moderate prevalence levels (30%), but the conditions for invasion are very broad when MK is common: then even highly costly suppression systems can spread. This itself leads to the conclusion that suppression can, on occasion, be a radical evolutionary change, one that would otherwise be prevented by a deep adaptive valley.

Many of the symbioses in which no suppression has been observed are low prevalence infections, for which the cost of suppression may exceed the benefit of rescuing males; some of them may additionally exhibit CI (CI may be masked where no suppression has evolved). However, not all suppressor-free systems are at low prevalence. In the butterfly *Acraea encedon,* MK *Wolbachia* is present in 95% of females [62]. Is there no available mutation that allows male host survival for all MK bacteria? If a mutation does exist, is it a complex mutational event (such as a rearrangement, a novel gene or a combination of linked mutations) and thus likely to be rare? The absence of suppression to high prevalence MK, as in *A. encedon*, implies some form of constraint. Thus one area of onward enquiry relates to the presence/absence of a constraint to the evolution of suppression. The conjecture here is that some host systems are less labile to change than others.

## 5. The dynamics of suppression

The fate of the suppressor following invasion again depends on whether the symbiont has other effects on host phenotype aside MK. Initial models presumed MK as the sole drive phenotype [60]. In this circumstance, the suppressor leads to loss of the drive phenotype, and thus a decline in the frequency of the symbiont. As the suppressor spreads, so the symbiont becomes less common. If suppression is cost free, the suppressor excludes the symbiont, and remains polymorphic in the population, evolving under genetic drift. If the suppressor carries a cost in the absence of the symbiont, an equilibrium is possible with the symbiont at lower frequency, and the suppressor remaining polymorphic. However, exclusion may occur if symbiont frequency is sufficiently reduced to make stochastic loss an impediment to persistence. In addition, damped cycles are possible, where the male-killer is reduced to very low frequency, followed by declines in suppressor frequency that eventually release the male-killer to spread again. Polymorphic suppression alongside polymorphic MK is apparent in the *Cheilomenes*/MK interaction [48].

While traditionally considered as having a single phenotype, we now recognize endosymbionts commonly have multiple phenotypic influences on their host, either concurrently or dependent on the context of the interaction. We know that MK can be combined with protective symbiosis [63,64]. We also know MK symbionts may have the capacity to exhibit CI when MK suppression rescues males as in *H. bolina* [41]. The dynamics of both MK suppression, and the symbiont, will depend on these alternate phenotypes. The emergence of CI following suppression has been modelled [61]. As argued above, the presence of CI can impede the initial invasion of a suppressor if MK is at low frequency. However, if MK frequency is high and the suppressor can invade, the CI phenotype continues to drive the symbiont to high frequency, and subsequently causes the suppressor to fix in the population. The population then appears as one carrying a CI *Wolbachia*, only revealing MK in crosses to other populations (e.g. [40]), or following trans-infection to a novel host species without a history of suppression (e.g. *w*CauA [53]).

The case in which MK symbionts have a second concurrent phenotype (that is not CI) has not been formally examined in the literature. Likely dynamics can nevertheless be inferred. Before the evolution of suppression, the symbiont would be at a prevalence that is a function of its MK phenotype plus its second benefit (e.g. natural enemy resistance). We can recognize two potential circumstances: (1) where the second benefit is in itself sufficient to maintain the symbiont, and (2) where the second phenotype must combine with the advantage of MK to overcome segregational loss. In the former case, the symbiont will be maintained even if the suppressor is cost free; a cost-free suppressor would fix, and a costly suppressor would rise to higher frequencies, reflecting the higher prevalence of the symbiont and continued threat from MK. Suppression would cause the symbiont frequency to decrease, but not to the extent observed where MK is the sole driver. By contrast, for a symbiont where the additional benefit is not sufficient for maintenance, elimination of the symbiont will still occur if the suppressor is cost free, and a reduction to very low prevalence is likely for suppressors with a modest cost.

## 6. Concluding remarks and future directions

Research into the mechanisms of MK is still in its infancy despite MK first being described over 70 years ago. In part, this has been a consequence of our inability to culture or genetically edit the vast majority of endosymbiotic bacteria, and likewise the ‘non-model’ status of many of the arthropods infected. It is notable that progress has been greatest in drosophilids, *Nasonia* and moth pests, all of which have good laboratory tractability. Recent progress has made it apparent that phylogenetically distant maternally inherited bacteria have independently evolved to selectively kill males of a wide variety of arthropods. The diversity in host sex determination systems, specificity of MK pathologies, and differences in the timing of male lethality support the conclusion that the MK phenotype is the result of convergent evolution [65]. While the overarching phenotype of MK is common—that infected males die early in development—the genetic, cellular and molecular mechanism of MK is dependent on the MK-host context.

We have seen that endosymbionts can specifically kill male offspring through direct manipulation of some component of the host's sex-determining cascade. In *Ostrinia* moths, MK acts prior to male-specific splicing of *dsx*, downregulating *Masc*, resulting in the prevention of dosage compensation and consequently male death. Male-specific death may also involve aspects of maleness outside the primary sex determination cascade; for MK *Spiroplasma* infecting *Drosophila,* it is the male-specific process of dosage compensation itself that is targeted. By contrast to these, the *Arsenophonus* son-killer appears to kill male wasps only indirectly; sexual identity is not targeted and the downstream male-specific sex determination pathways are unaffected. In terms of microbial factors, putative MK genes have recently been reported for both *Spiroplasma* (*spaid*) and *Wolbachia* (*wmk*), but the manner in which these genes effect male-specific death is still unclear, and their function in natural systems is still to be confirmed. Understanding the mechanism of both MK and host suppression of MK more fully is likely to illuminate the evolutionary biology of these interactions.

The mechanistic basis of suppression is, to date, uncharacterized. That male-killers target aspects of sex determination and ‘collateral systems' such as dosage compensation imply that these pathways are likely to be those in which the evolutionary response of suppression acts. To understand the degree to which male-killers drive sex determination diversity requires us to understand what fraction of male-killers are primarily targeting the core pathways in these systems (in contrast to *Arsenophonus* MK). A mechanism would need to be established for diverse male-killers and, given the range of sex determination systems, diverse host species. While our understanding of the mechanism of MK caused by *Wolbachia* and *Spiroplasma* in laboratory systems is increasing, we currently know very little about MK induced by *Rickettsia*, Flavobacteria, the Gammaproteobacteria infecting *C. sexmaculata,* or indeed viruses. We also know little of the molecular basis of MK caused by *Wolbachia* and *Spiroplasma* infecting butterflies and ladybirds, despite MK being relatively common in these groups.

In addition to understanding which features of insect biology evolve in response to male-killers, understanding the MK mechanism is also important in recognizing the conditions under which the evolution of suppression is constrained. Part of this we know—for instance, the capacity of a symbiont to additionally produce CI inhibits suppressor spread for low prevalence MK. However, part of the constraint is likely to be mechanistic, where the target is a feature of biology/development that is very hard to alter while retaining function.

These considerations motivate a research programme where the interaction of diverse MK bacteria are examined, alongside a diverse set of suppression systems. In particular, it is important to analyse MK alongside suppression wherever possible. This is not a simple endeavour—these are commonly non-model insects. Nevertheless, work on non-models is becoming more feasible. Additionally, progress may be enabled using cell culture models. Although isolated cultivation of most heritable symbionts is not successful [66], *Wolbachia* at least can be cultivated by *in vitro* cell culture (reviewed in [67]) or *ex vivo* organ culture [68], and other heritable symbionts can also be maintained in this way. By this process, it has recently been discovered that in cell lines established from *O. scapulalis* males, the splicing pattern of *dsx* can be altered from male-type to female-type by MK *Wobachia* but not by non-MK *Wolbachia*, recapitulating the pattern seen within the insect. Considering that *w*CauA does not cause MK in its native host *E. cautella* but does cause MK in the novel host *E. kuehniella*, a cell culture system would be useful to identify the MK ability of *Wolbachia* even where it is masked by suppressors in their native hosts.

In summary, it is becoming clear that MK endosymbionts interact directly, or indirectly, with the host's sex determination mechanism in a myriad of ways, dependent on both male-killer and host identity. We know from observation that host suppression of MK activity can be very strongly selected, making it feasible that suppression may include components of the sex-determination system that are normally not subject to evolutionary change. Endosymbionts, particularly male-killers, have been proposed to exert the selective cost necessary to drive an ‘arms race’ between them. We therefore conjecture that the host's response to MK microbes may represent an unrecognized driver of the diversity of arthropod sex determination and allied processes.

## Data Availability

The data are provided in electronic supplementary material [69].
